# Intake of oligoelements with cytarabine or etoposide alters dopamine levels and oxidative damage in rat brain

**DOI:** 10.1038/s41598-024-61766-0

**Published:** 2024-05-12

**Authors:** David Calderón Guzmán, Norma Osnaya Brizuela, Maribel Ortíz Herrera, Hugo Juárez Olguín, Armando Valenzuela Peraza, Norma Labra Ruíz, Gerardo Barragán Mejía

**Affiliations:** 1 Laboratory of Neurosciences, Instituto Nacional de Pediatria, Mexico City, Mexico; 2 Laboratory of Experimental Bacteriology, Instituto Nacional de Pediatria, Mexico City, Mexico; 3 Laboratory of Pharmacology, Instituto Nacional de Pediatria, Av. Iman No.1, 3er piso, Col. Cuicuilco, 04530 Mexico City, CP Mexico; 4 Department of Pharmacology, Universidad Nacional Autónoma de Mexico, Mexico City, Mexico

**Keywords:** Brain, Cytarabine, Etoposide, Oligoelements, Oxidative damage, Cancer, Neuroscience

## Abstract

Research on the relationships between oligoelements (OE) and the development of cancer or its prevention is a field that is gaining increasing relevance. The aim was to evaluate OE and their interactions with oncology treatments (cytarabine or etoposide) to determine the effects of this combination on biogenic amines and oxidative stress biomarkers in the brain regions of young Wistar rats. Dopamine (DA), 5-Hydroxyindoleacetic acid (5-Hiaa), Glutathione (Gsh), Tiobarbituric acid reactive substances (TBARS) and Ca^+2^, Mg^+2^ ATPase enzyme activity were measured in brain regions tissues using spectrophometric and fluorometric methods previously validated. The combination of oligoelements and cytarabine increased dopamine in the striatum but decreased it in cerebellum/medulla-oblongata, whereas the combination of oligoelements and etoposide reduced lipid peroxidation. These results suggest that supplementation with oligoelements modifies the effects of cytarabine and etoposide by redox pathways, and may become promising therapeutic targets in patients with cancer.

## Introduction

**Figure 1:**
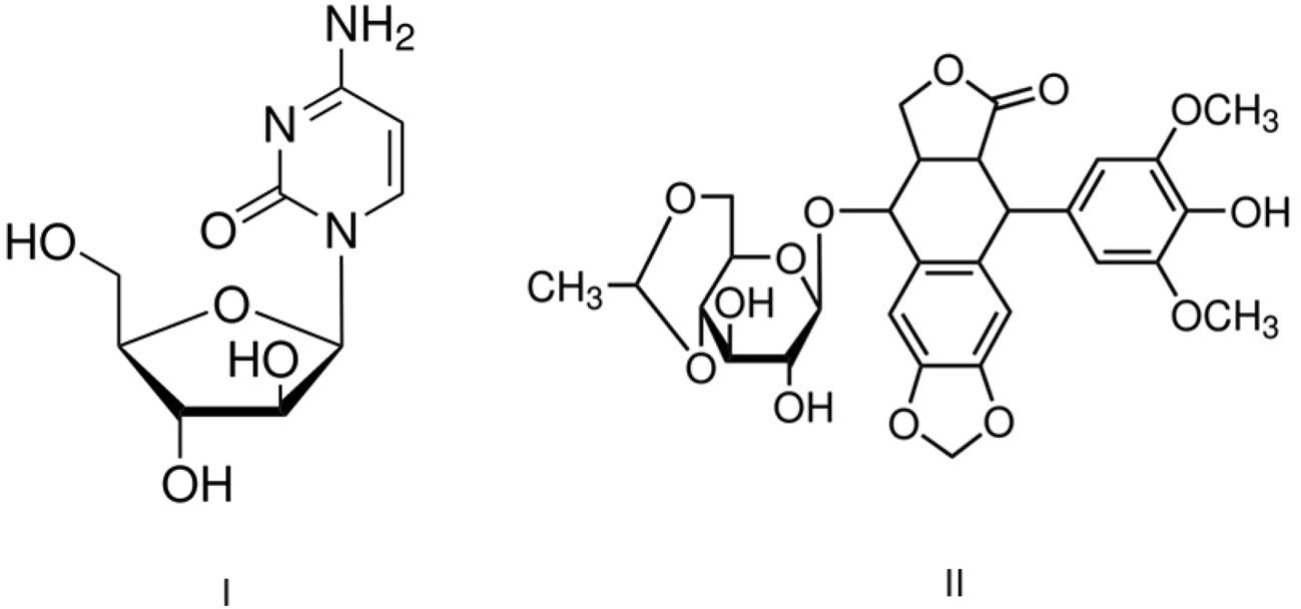
Cytarabine (I) and Etoposide (II) chemical structures.

Cancer death toll around the world has toppled all causes leading to loss of life. Annual new cases**,** due to this ailment, have surpassed ten million people, while death registry is estimated to be over six million. Yildiz et al.^1^, found that more than 80% of the cancers, which the people suffer side effects**,** and in United States, malignant tumors are currently the main cause of death in children under 15 years of age^2^. Cytarabin and etoposide (Fig. 1) are the drugs extensively employed in the treatment of cancer in pediatric population and high doses has been used to treat hematologic malignancies^3^. The mechanisms of these drugs are thought to be mediated by oxidative stress^4–6^, and the dysfunction of mitochondrial respiratory chain induced by chemotherapy agent results in overproduction of reactive oxygen species (ROS)^7^, and then free radicals that lead to the onset of oxidative stress are detrimental to the cells, and cause protein denaturation, lipid peroxidation and DNA structural damage^8–10^.

Cell membrane is composed by different types of lipids, and modifications in this cell structure can affect many biological processes^11^. In the brain, the phospholipids that compose the membrane of the plasma are contiguous with the protein architecture inside the double strand lipid layers of the membrane^12^. The interchange of ions taking place in these double strand lipid layers is facilitated by Na^+^, K^+^ ATPase enzyme that stimulates the entrance and exit of Na^+^ and K^+^ in the cell^13^. ATPase is a membrane protein whose activity in the central nervous system (CNS) is very sensitive to oxidative stress^14,15^. Enzymatic activity modification of Na(+)/K(+) ATPase has been reported in anti-cancer agents induced oxidative stress^16^. Also, alteration in monoamines as dopamine and serotonin in the CNS results from the administration of oncology drugs^17,18^. These adverse effects may be alleviated by administration of antioxidant drugs or oligoelements (OE). These elements have been shown to exert considerable clinical benefits^19–21^. Research on the relationships between trace elements and the development of cancer or its prevention is a field that is gaining increasing relevance due OE play a catalytic role in enzyme systems^22^. For example, iron and copper participate in oxidation–reduction reactions in energy metabolism; and zinc participates in nucleic acid metabolism, cell replication and growth through its function in nucleic acid polymerases^23^. However, OE also play a very important role on cancer development. Therefore, the impairment of certain neurochemical processes due to shortage or excess quantity of trace elements may have brain consequences^24^. Increase in serum levels of Co, Cu, Mg and Pb was found in patients with prostate cancer^25^. Differences in the concentration of Cu, Zn and Se in malignant tissue have been reported^26^. On the other hand, trace elements also play an important role in antineoplastic treatment. According to Liu et al.^27^, selenium and manganese content in primary cancer tissues could influence the response of the cells to carboplatin and doxorubicin. Various form of cancer is comprised of a subpopulation of malignant cells and the regions where this is most relevant is within the brain^28^. Therefore, the objective of this study was to evaluate the intake or supplementation of OE with etoposide and cytarabine on selected biogenic amines and on oxidative stress markers in the brain regions of young rats.

### Chemicals

Thiobarbituric acid (TBA), Glutathione, ATP and 5-HIAA were obtained from Sigma-Aldrich, St. Louis, MO, USA. Hydrochloric acid, Sulfuric acid, Nitric acid, Bisulfite, Trichloro acetic acid, Sodium phosphate and Magnesium chloride, Tris–HCl were purchased from Merck, Darmstad, Germany.

## Experiments

Thirty-six female Wistar rats**,** 4 weeks old (mean weight 70 g)**,** were procured from Bioterium of Metropolitan University of Mexico City and housed six per cage in clean plastic cages in a room with a 12-h light:12-h dark cycle at 22 ± 2 °C with a relative humidity of 50 ± 10%. Tekla Rodent diet 5001 (protein 23%) and drinking water were given to the animal’s ad libitum.

Animals were randomly assigned to 6 groups and treated as follows: control (200 µl NaCl 0.9%,), cytarabine (0.08 mM), etoposide (0.007 mM), Oligoelements mix (50µL), oligoelements + etoposide and oligoelements + cytarabine. All treatments were administered via intraperitoneal injection.

The oligoelements administered is 10-mL ampoule containing 20 µmol of iron (1.1 mg FeCl_2_ × 6H_2_O), 77 µmol of zinc (5 mg of ZnCl_2_), 1 µmol of manganese (55 µg of MnCl_2_ × 4H_2_O), 1 µmol of selenium (79 µg Na_2_SeO_3_), 0.2 µmol of chromium III (10 µg CrCl_3_ × 6H_2_0), 6 µmol of copper (380 µg CuCl_2_ × 2H_2_O), 0.2 µmol of molybdenum (19 µg Na_2_MoO_4_ × 2H_2_O), 1 µmol of iodine (127 µg KI) and 50 µmol of fluorine (950 µg NaF).

Oligoelements (Nulanza®) were obtained from Fresenius Kabi. Cytarabine® was procured from Pfizer and Etoposide® from Bristol-Myers Squibb.

At the end of the experiment, the animals were euthanized by decapitation without anesthesia. The brains were extracted and immediately placed in a 0.9% cold NaCl solution. Brains were anatomically dissected in cortex, striatum and cerebellum/medulla-oblongata and homogenized in 0.05 mol L^–1^ phosphate buffer solution (PBS). The samples were frozen at –20 °C until analysis. Measurements of thiobarbituric acid reactive substances (TBARS), Ca^+2^, Mg^+2^ ATPase activity, glutathione (GSH), serotonin metabolite (5-Hiaa) and dopamine were carried out using spectrophotometric and fluorometric methods^29^. Only this select biomarkers were measured due tissue sample size is limited by assays.

### Statistical analysis

Analysis of variance (ANOVA) and Kruskal–Wallis test were used with post hoc comparisons after homogeneity variance verification. Values of *p* < 0.05 were considered statistically significant. For data analysis, the JMP v12 software (SAS Institute, USA) was used. All methods are reported in accordance with ARRIVE guidelines.

### Ethical approval

Animal experiments were carried out under strict compliance with the Guidelines for Ethical Control and Supervision in the Care and Use of Animals, and all experimental procedures were done following national and international rules. The study was approved by committees of Instituto Nacional de Pediatria (Code 026/2022).

## Results

### Dopamine (DA)

Dopamine concentration experimented a slight decrease in the cortex of all the treatment groups when compared with the control group; however, this decrease was not statistically significant.

In the striatum, the treatment with OE + cytarabine induced significant increase in the concentration of DA when compared with the control (*p* < 0.0001), OE (*p* < 0.0001), cytarabine (*p* < 0.0005) and OE + etoposide (*p* < 0.0008).

An opposite effect was observed in cerebellum/medulla-oblongata. DA concentration in the group of animals treated with OE + cytarabine decreased significantly (*p* < 0.005) with respect to control, OE (*p* < 0.008), cytarabine (*p* < 0.02) and OE + etoposide (*p* < 0.005). Administration of OE + Etoposide induced an increase in DA on comparing this increase with what happened with etoposide treated group (*p* < 0.05) (Table 1).

**Table 1:** Dopamine concentration (nmol g^−1^) in brain of rats treated with of Nulanza in combination with Cytarabine or Etoposide.

Dopamine (nmol g^−1^)			
Treatment	Cortex	Striatum	Cerebellum-medulla-oblongata
Control	158.11 ± 47.88	138.39 ± 39.03	297.02 ± 79.91
Nulanza	162.92 ± 46.51	101.25 ± 21.10	255.05 ± 78.14
Cytarabine	138.43 ± 45.08	145.53 ± 26.66	175.52 ± 93.22*
Etoposide	123.42 ± 17.33	138.67 ± 29.56	198.11 ± 25.55
Nulanza + Cytarabine	134.92 ± 42.41	247.67 ± 32.03*	60.86 ± 26.15**
Nulanza + Etoposide	132.45 ± 41.66	150.48 ± 45.47	275.74 ± 66.73

OE + cytarabine increased dopamine in the striatum: Anova * *p* < 0.0002, **p < 0005, ***p < 0.0008; but decreased it in cerebellum/medulla-oblongata: Kruskal–Wallis *p < 0.005, **p < 0.02, ***p < 0.05, ****p < 0.008, *****p < 0.005.

### 5-Hydroxyindole acetic acid (5-HIAA)

Analysis of the data revealed no effect on 5-HIAA concentrations attributable to any of the treatments (Data not shown).

### Glutathione (GSH)

In the cortex, the animals treated with cytarabine showed an increase in GSH level in comparison with control (*p* < 0.05), and with those treated with either OE alone (*p* < 0.0001) or only etoposide (*p* < 0.003). Comparison between cytarabine versus OE + cytarabine showed a significant decrease in DA concentration for OE + etoposide group (*p* < 0.0001). GSH levels in OE + etoposide group was found significantly lower when compared with etoposide alone (*p* < 0.006) and the control (*p* < 0.001).

In striatum and cerebellum/medulla-oblongata, GSH levels were reduced in all the animal groups when compared with the control group; however, the statistical analysis revealed thatthe reduction was significant (*p* < 0.05) only in the cytarabine treated group. In cerebellum/medulla-oblongata, no difference in GSH levels was found (Table 2).

**Table 2:** Levels of Glutathion (GSH) (nmol g^−1^) in brain of rats treated with Nulanza in combination with Cytarabine or Etoposide.

GSH (nmol g^−1^)			
Treatment	Cortex	Striatum	Cerebellum-medulla oblongata
Control	64.50 ± 8.81	80.11 ± 7.44	60.50 ± 17.33
Nulanza	48.52 ± 20.12	66.48 ± 24.75	45.54 ± 14.88
Cytarabine	92.87 ± 6.50*	53.43 ± 12.09*	62.97 ± 12.72
Etoposide	57.91 ± 20.44	68.55 ± 16.73	51.99 ± 7.71
Nulanza + Cytarabine	44.40 ± 15.45	63.11 ± 13.02	44.83 ± 5.30
Nulanza + Etoposide	32.09 ± 8.20**	55.98 ± 23.79	51.48 ± 7.34

Animals treated with cytarabine showed increased GSH levels in cortex: Anova *p < 0.05**p < 0.003, while a decrease was observed for OE + etoposide group ***p < 0.006 versus etoposide. Reduction in GSH in striatum was observed for cytarabine treated group *p < 0.05. No significant differences were found in cerebellum/medulla-oblongata.

### Lipid peroxidation (TBARS)

The analysis of lipid peroxidation by-products in the cortex region revealed that the animals that received cytarabine alone and OE + etoposide showed a significant decrease in the concentration of this oxidative stress marker with respect to the control group (*p* < 0.05). Data analysis did not show any difference in concentration of TBARS for the other groups of treatment.

In the striatum, a decrease in TBARS was observed in all the experimental groups in comparison with the control, but only cytarabine and OE + etoposide showed to be statistically different (*p* < 0.01).

Concentrations of TBARS in cerebellum/medulla-oblongata were comparable with that found in the cortex. The animals that received cytarabine alone and the combination of OE + etoposide showed a significant decrease when compared with the control (*p* < 0.02). Also, the comparison of the level of TBARS for cytarabine versus OE and for OE versus OE + etoposide showed to be significant *p* < 0.008 and *p* < 0.02 respectively (Table 3).

**Table 3:** Lipid peroxidation (TBARS) (µmol malondialdehyde g^−1^) in brain of rats treated with Nulanza in combination with Cytarabine or Etoposide.

Tbars (µmol malondialdehyde g^−1^)			
Treatment	Cortex	Striatum	Cerebellum-medulla oblongata
Control	7.69 ± 1.70	12.26 ± 1.39	8.62 ± 0.63
Nulanza	6.36 ± 1.42	8.48 ± 1.62	8.32 ± 1.67
Cytarabine	5.16 ± 0.97*	7.25 ± 1.97*	5.40 ± 0.54*^a^
Etoposide	7.40 ± 2.20	9.49 ± 3.01	7.47 ± 2.14
Nulanza + Cytarabine	6.18 ± 1.58	9.80 ± 3.19	7.44 ± 1.92
Nulanza + Etoposide	5.31 ± 1.00*	7.23 ± 2.12*	5.35 ± 0.82*^a^

Cytarabine or OE + etoposide decreased lipid peroxidation in cortex: Anova *p < 0.05, **p < 0.02. In striatum and cerebellum/medulla-oblongata, we observed reduced lipid peroxidation by cytarabine and OE + etoposide *p < 0.01; and OE versus OE + etoposide Kruskal–Wallis **p < 0.02.

### Ca^+2^, Mg^+2^ ATPase

No major changes were observed in the activity of this enzyme in the cortex of the experimental animals. However, in the striatum, when the groups treated with cytarabine and OE + cytarabine were compared with the control group, the activity of this enzyme was found significantly increased (*p* < 0.008). In addition, significant increase of the enzyme activity was observed in OE + cytarabine group when compared with those that received only OE (*p* < 0.02). An opposite effect was seen in the administration of OE + cytarabine or OE + etoposide (*p* < 0.02).

In cerebellum/medulla-oblongata, the groups that received cytarabine alone and OE + cytarabine depicted a very high activity of the enzyme that resulted in an increase with significantly statistic difference (*p* < 0.008) when compared with the control (*p* < 0.0005). Comparison between OE versus OE + cytarabine groups showed a significant increase (*p* < 0.02). In addition, significant differences were observed between the group of animals treated with the combination OE + cytarabine and OE + etoposide (*p* < 0.01) (Table 4).

**Table 4:** Activity of Na+, K+ATPase enzyme (µmol Pi g^−1^ min^−1^) in brain of rats treated with Nulanza in combination with Cytarabine or Etoposide.

Ca^+2^, Mg^+2^ ATPase (µmol Pi g^−1^ min^−1^)			
Treatment	Cortex	Striatum	Cerebellum-medulla oblongata
Control	385.70 ± 68.23	578.12 ± 146.67	789.40 ± 47.30
Nulanza	396.85 ± 30.34	690.10 ± 112.23	856.23 ± 93.75
Cytarabine	415.04 ± 101.93	910.33 ± 204.02*	1152.08 ± 262.09*
Etoposide	370.77 ± 71.48	713.67 ± 133.55	843.81 ± 91.83
Nulanza + Cytarabine	455.44 ± 170.23	1007.58 ± 187.28*^a^	1282.99 ± 207.38*^a^
Nulanza + Etoposide	486.31 ± 184.52	803.96 ± 90.85	970.89 ± 92.66

Increased ATPase activity in striatum was observed as a result of cytarabine and OE + cytarabine treatments as compared to the control Anova *p < 0.008 and OE group *p* < 0.02. Opposite effect resulted from OE + cytarabine or OE + etoposide administration **p < 0.02. In cerebellum/medulla-oblongata, the group treated with cytarabine or OE + cytarabine showed a very high activity when compared with the control Kruskal–Wallis *p < 0.01 or OE group *p* < 0.02. Once again, opposite effect resulted from OE + cytarabine or OE + etoposide administration **p < 0.02.

## Discussion

OE are required by man in amounts ranging from 50 µg to 18 mg per day. Acting as catalytic or structural components of larger molecules, they have specific functions^30^, and therefore OE their dietary interactions and their main food sources can provide patients quality of life and therapy outcomes^31^.

In the present study, the administration of OE or etoposide alone showed no significant effects on the selected parameters. Cytarabine administration produced important changes in dopamine, glutathione and Ca^+2^, Mg^+2^ ATPase; when combined with OE, cytarabine increases the concentration of DA in the striatum and reduces this biomarker in the cerebellum/medulla-oblongata. We also observed an increase in Ca^+2^, Mg^+2^ ATPase activity in the striatum and cerebellum/medulla-oblongata as a result of the administration of OE + cytarabine. These effects in brain regions may be due reactive oxygen species (ROS)-inducing cytarabine^18^. In fact, mitochondria are key regulators of cell survival such as metabolism, Ca^2+^ signaling ROS production. However; ROS overproduction and mitochondrial Ca^2+^ overload are linked to the induction of apoptosis, while the impairment of mitochondrial dynamics and metabolism can have a double-faceted role in the decision between cell survival and death^32^. Indeed, chemoresistance, which may be due to the cooperation of several cellular protection mechanisms, often mitochondria-related.

Treatment with OE + etoposide depicted a more evident effect on glutathione and lipid peroxidation. A pronounced decrease in GSH levels was observed in the cortex, while TBARS reduction was observed in the brain regions. These findings suggest that OE play a catalytic role in enzyme systems and activate the antioxidant role of etoposide^33^. However; different cancer cell types may undergo different bioenergetic changes, some to more glycolytic and some to more oxidative. The energy powerhouse of a cell, represent key intracellular signaling hub that are emerging as important determinants of several aspects of cancer development and progression, including metabolic reprogramming, acquisition of metastatic capability, and response to chemotherapeutic drugs^34^. The brain is a major metabolizer of oxygen, it has relatively feeble protective antioxidant mechanisms. Therefore, the modulation of the prooxidant-antioxidant balance provides a therapeutic option, which can be used to improve neuroprotection in response to oxidative stress^35^. This finding coincide with our results, due seem that OE supplementation appears to improve the effects of anticancer drugs. Indeed, trace elements can be used as not only a preventive but also a therapeutic tool, especially in reducing inflammation in hematological cancers as suggest Jahankhani et al.^36^.

Tumor cells are characterized by substantial changes in their metabolism thus affecting the need for micronutrients. Besides, these compounds are suitable activators of antioxidant enzymes on redox endogenous system for cellular homeostasis^37^, and this type of study may help to developmental neurobiology that have uncovered new perspectives from which it investigate various forms of cancer.

Recent studies with OE and anticancer agents suggest that the cytarabine molecule undergoes hydroxylation and subsequent oxidation after its administration^38^. According to Tan et al.^39^, sodium selenite accentuates the therapeutic potential of adriamycin prodrug in gastric cancer treatment. Xue et al.^40^ reported that zinc in combination with paclitaxel (PXT) inhibits the invasion and migration of prostate cancer cells and increases the sensitivity of prostate cancer cells to PTX, while Lee et al.^41^, demonstrated that supplement of copper significantly enhances the inhibitory effect of curcumin on oral cancer cells. These findings support the idea that supplementation with OE during chemotherapy can improve the effectiveness of drugs, and then adjunctive therapy based on dietary supplement, which would facilitate the improvement of antioxidant status and re-establishment of tissue GSH, may be developed.

On the other way, in the present study ara-C (cytarabine) induced toxicity and could be seen as biphasic phenomenon, due to the permeability transition occurring after a depletion of GSH and preceding a state of high reactive oxygen generation.

Probably *O*-demethylation of etoposide to etoposide catechol (etoposide-OH) by cytochrome P450 3A4 (CYP3A4) may have formed etoposide semi-quinone (etoposide-SQ) and etoposide quinone (etoposide-Q) that are known to react with glutathione to produce oxidative damage^42^. However, etoposide acts as an effective radical scavenger and antioxidant protector of intracellular phospholipids ^43^.

Cytarabine administration increased dopamine concentration as depicted by the result of this study, due it drug is highly effective in preventing the death of postmitotic dopaminergic neurons that occurs spontaneously by apoptosis^44^, and dopamine higher is associated with elevated oxidative damage^45^. In fact, previous studies suggest that dopamine receptors may serve as a biomarker for diverse malignancies^46^. Although novel analogues may improve the potential to repurpose this class of drugs to treat brain tumors and/or brain metastasis^47^. Finally, is important knowledge that dopamine changes in brain regions could have implications for motor control, modulation of affective and emotional states, reward mechanisms, reinforcement of behavior, and selected higher cognitive functions^48^. However; OE supplementation probably exacerbated cytarabine effects by the elevation of ROS production in this study. Despite that OE are essential for antioxidant enzymes to function properly, or excess of them can also act as pro-oxidants, due marginal or severe trace element imbalances can be considered risk factors for several diseases^30^.

## Conclusions

The efficacy of combined OE and Cytarabine or etoposide drug therapy appears to be a promising strategy for future chemotherapy in young patients. We suggest that more research work be carried out to thoroughly examine its neuroprotective mechanisms. However further investigations are needed to validate the beneficial or detrimental effects of OE administration in cancer treatment using different biochemical oxidative damage markers.

## Data Availability

Any data and material used in this study are available on request to the correspondence author.
